# Correction: Exploring scalable assessment methods for terminated trials in ClinicalTrials.gov: A cohort analysis of German and Californian trials

**DOI:** 10.1371/journal.pone.0352277

**Published:** 2026-06-22

**Authors:** Samruddhi Suresh Yerunkar, Benjamin Gregory Carlisle, Delwen L. Franzen, Maia Salholz-Hillel, Daniel Strech, Susanne Gabriele Schorr

S1 File was uploaded incorrectly. Please see the correct S1 File below.

## Supporting information

S1 File Supporting information file: Includes details about Description: Degree of enrolment – Handling of missing enrollment variable. Table. Reason for termination – categorization. Description: SAE risk analysis methodology and PRISMA flow diagram. Table. Detailed therapeutic foci table5 Table. Trial characteristics by reason for termination.
(DOCX)
