# Supplementary material for: Exploring scalable assessment methods for terminated trials in ClinicalTrials.gov: A cohort analysis of German and Californian trials
Source: PLoS One. 2026 Apr 15;21(4):e0346608. doi: 10.1371/journal.pone.0346608 (PMC13082632; doi:10.1371/journal.pone.0346608)
Supplement: Peer review history

# Peer review: Decision Letter 0

**DOI:** 10.1371/journal.pone.0346608.r001

**Authors:** Sascha Köpke

7 Dec 2025

Dear Dr. Yerunkar,

Thank you for submitting your manuscript to PLOS ONE. After careful consideration, we feel that it has merit but does not fully meet PLOS ONE’s publication criteria as it currently stands. Therefore, we invite you to submit a revised version of the manuscript that addresses the points raised during the review process.

Please submit your revised manuscript by Jan 21 2026 11:59PM. If you will need more time than this to complete your revisions, please reply to this message or contact the journal office at plosone@plos.org. When you’re ready to submit your revision, log on to https://www.editorialmanager.com/pone/ and select the ‘Submissions Needing Revision’ folder to locate your manuscript file.. When you’re ready to submit your revision, log on to https://www.editorialmanager.com/pone/ and select the ‘Submissions Needing Revision’ folder to locate your manuscript file.. When you’re ready to submit your revision, log on to https://www.editorialmanager.com/pone/ and select the ‘Submissions Needing Revision’ folder to locate your manuscript file.. When you’re ready to submit your revision, log on to https://www.editorialmanager.com/pone/ and select the ‘Submissions Needing Revision’ folder to locate your manuscript file.

- A rebuttal letter that responds to each point raised by the academic editor and reviewer(s). You should upload this letter as a separate file labeled ‘Response to Reviewers’.
- A marked-up copy of your manuscript that highlights changes made to the original version. You should upload this as a separate file labeled ‘Revised Manuscript with Track Changes’.
- An unmarked version of your revised paper without tracked changes. You should upload this as a separate file labeled ‘Manuscript’.

If you would like to make changes to your financial disclosure, please include your updated statement in your cover letter. Guidelines for resubmitting your figure files are available below the reviewer comments at the end of this letter.

We look forward to receiving your revised manuscript.

Kind regards,

Sascha Köpke

Academic Editor

PLOS One

Journal Requirements:

When submitting your revision, we need you to address these additional requirements.

1. Please ensure that your manuscript meets PLOS ONE’s style requirements, including those for file naming. The PLOS ONE style templates can be found at https://journals.plos.org/plosone/s/file?id=wjVg/PLOSOne_formatting_sample_main_body.pdf and https://journals.plos.org/plosone/s/file?id=ba62/PLOSOne_formatting_sample_title_authors_affiliations.pdf

2. Please upload a new copy of Figure 1 as the detail is not clear. Please follow the link for more information: https://journals.plos.org/plosone/s/figures

3. Please include captions for your Supporting Information files at the end of your manuscript, and update any in-text citations to match accordingly. Please see our Supporting Information guidelines for more information: http://journals.plos.org/plosone/s/supporting-information.

4. If the reviewer comments include a recommendation to cite specific previously published works, please review and evaluate these publications to determine whether they are relevant and should be cited. There is no requirement to cite these works unless the editor has indicated otherwise.

[Note: HTML markup is below. Please do not edit.]

Reviewers’ comments:

Reviewer’s Responses to Questions

**Comments to the Author**

1. Is the manuscript technically sound, and do the data support the conclusions?

Reviewer #1: Yes

Reviewer #2: Yes

**********

2. Has the statistical analysis been performed appropriately and rigorously?

Reviewer #1: No

Reviewer #2: Yes

**********

3. Have the authors made all data underlying the findings in their manuscript fully available?

The PLOS Data policy requires authors to make all data underlying the findings described in their manuscript fully available without restriction, with rare exception (please refer to the Data Availability Statement in the manuscript PDF file). The data should be provided as part of the manuscript or its supporting information, or deposited to a public repository. For example, in addition to summary statistics, the data points behind means, medians and variance measures should be available. If there are restrictions on publicly sharing data—e.g. participant privacy or use of data from a third party—those must be specified.requires authors to make all data underlying the findings described in their manuscript fully available without restriction, with rare exception (please refer to the Data Availability Statement in the manuscript PDF file). The data should be provided as part of the manuscript or its supporting information, or deposited to a public repository. For example, in addition to summary statistics, the data points behind means, medians and variance measures should be available. If there are restrictions on publicly sharing data—e.g. participant privacy or use of data from a third party—those must be specified.

Reviewer #1: Yes

Reviewer #2: Yes

**********

4. Is the manuscript presented in an intelligible fashion and written in standard English?

Reviewer #1: Yes

Reviewer #2: Yes

**********

Reviewer #1: 1. Please define cleary the eligibility criteria used in CT.gov registry. It is not defined did you retrieved trials with or without results, and which phases are included. Please clearly define that in “Eligibilty criteria” section” and in the data in OSF platform. The inclusion criteria should be defined following the filters in the ClinicalTrials.gov registry.

2. Exclusion criteria is not defined also. This should be defined in the part “Eligibility criteria” section of manuscript.

3. Differences in the ClinicalTrials.gov and other registries in the variety of trials should be presented as a limitation and explaines in that part of manuscript.

Reviewer #2: Thank you for the opportunity to review this interesting article about terminated trials from German and Californian university medical centers identified via ClinicalTrials.gov and the German study registry.

The data analysis consists of two previously assembled cohorts of clinical trials registered in California and Germany. The study explores the characteristics of prematurely terminated clinical trials affiliated with university medical centers in aforementioned locations, focusing on developing scalable semi-automated methods for their assessment. The authors compare terminated trials to completed ones regarding characteristics like result reporting and therapeutic focus. A secondary, exploratory aim was to estimate the risk of experiencing serious adverse events (SAEs) in trials terminated for non-scientific reasons.

Overall, the manuscript is well written, minor issues that could be addressed are as follows:

- Centres and centers could be written consistently in one form or the other.

- Non-scientific and nonscientific should be written consistently

Minor issues:

Abstract: In the abstract, the authors describe the usage of the EUCTR as a data source in case of missing data in the ClinicalTrials register for the German studies; however, the authors do not mention that one primary data source for the German studies seems to be the DRKS database. If this is correct, please mention the database in the Abstract section; if it is not correct, please correct the methods so abstract and method sections are consistent.

Major issues:

- The manuscript states that publications for both cohorts were ‘searched and validated manually’, but no information is provided on how this search was conducted (databases used, search strings, matching criteria, number of reviewers, or cutoff date). As the analysis relies on publication detection originating from earlier cohort studies, the manuscript should summarise the original search methodology to ensure reproducibility and assess potential differences in search sensitivity between the German and Californian datasets.

- What was the reason the authors used a different time interval as a comparison between the German cohort (2009-2017) and the Californian cohort (2014-2017); this introduces a potential bias in the comparison and should be discussed.

- In general, potential biases and weaknesses of the study approach should be discussed in more detail: Slow enrollment leading to trial termination might both be due to (underlying) business reasons; e.g., insufficient financial incentives for the medical centers to recruit patients or due to low efficacy and/or toxicity. I.e., toxicity or low efficacy in the first few patients recruited by a center or physician could lead to reluctance by physicians to recruit further patients. Therefore business reasons and scientific reasons might overlap in many cases and it is not possible to systematically account for these issues.

Similarly, excluding journal articles as a source for SAE reporting introduces a systematic bias: Data on inconsistent reporting as cited by the authors are credible in cases in which there is both a ClinicalTrials information (on SAE) available and a publication. However, excluding trials from such an analysis in which the ClinicalTrials data were unavailable but a full Journal article introduces another bias as there might be a systematic difference between trial sponsors who report results on ClinicalTrials and sponsors who are more reluctant (but would publish results in journal articles). As an example: Industry sponsors reported on trials more promptly and adhered closer to legal obligations than academic trials (Anderson et al. NEJM 2015). An academic sponsor might be interested in a publication while an update on ClinicalTrials might be sufficient for an industry sponsor if there is no commercial viability of the tested approach.

- There are only 14 patients included in the exploratory analysis; however, the authors state that the analysis “revealed no difference in risk” between the intervention and the control group. This should be softened to a statement acknowledging the limitations of such a small sample size especially considering the multitude of weaknesses of such studies as mentioned above.

**********

what does this mean?). If published, this will include your full peer review and any attached files.). If published, this will include your full peer review and any attached files.). If published, this will include your full peer review and any attached files.). If published, this will include your full peer review and any attached files.

If you choose “no”, your identity will remain anonymous but your review may still be made public.

**Do you want your identity to be public for this peer review?** For information about this choice, including consent withdrawal, please see our For information about this choice, including consent withdrawal, please see our For information about this choice, including consent withdrawal, please see our For information about this choice, including consent withdrawal, please see our Privacy Policy..

Reviewer #1: No

Reviewer #2: No

**********

[NOTE: If reviewer comments were submitted as an attachment file, they will be attached to this email and accessible via the submission site. Please log into your account, locate the manuscript record, and check for the action link “View Attachments”. If this link does not appear, there are no attachment files.]

To ensure your figures meet our technical requirements, please review our figure guidelines: https://journals.plos.org/plosone/s/figures

You may also use PLOS’s free figure tool, NAAS, to help you prepare publication quality figures: https://journals.plos.org/plosone/s/figures#loc-tools-for-figure-preparation.

NAAS will assess whether your figures meet our technical requirements by comparing each figure against our figure specifications.

# Author comment: Author response to Decision Letter 1

**DOI:** 10.1371/journal.pone.0346608.r002

27 Feb 2026

Dear editor, Dear reviewers,

We are very grateful for the careful and constructive assessment of our manuscript. We appreciate the opportunity to revise and resubmit it. Please find our responses to the comments below:

Responses to the comments made by the editor:

Comment: Please ensure that your manuscript meets PLOS ONE’s style requirements, including those for file naming. The PLOS ONE style templates can be found at https://journals.plos.org/plosone/s/file?id=wjVg/PLOSOne_formatting_sample_main_body.pdf and https://journals.plos.org/plosone/s/file?id=ba62/PLOSOne_formatting_sample_title_authors_affiliations.pdf

Response: We have revised the manuscript and file naming to comply with PLOS ONE’s style requirements and templates.

Comment: Please upload a new copy of Figure 1 as the detail is not clear. Please follow the link for more information: https://journals.plos.org/plosone/s/figures

Response: The Figure 1 forest plot was re-generated in R and exported as vector-based EPS file matching the journal’s figure requirement.

Comment: Please include captions for your Supporting Information files at the end of your manuscript, and update any in-text citations to match accordingly. Please see our Supporting Information guidelines for more information: http://journals.plos.org/plosone/s/supporting-information.

Response: The Supporting Information captions have been included at the end of the manuscript, and the in-text citations have been updated accordingly to ensure consistency with the journal guidelines.

Comment: If the reviewer comments include a recommendation to cite specific previously published works, please review and evaluate these publications to determine whether they are relevant and should be cited. There is no requirement to cite these works unless the editor has indicated otherwise.

Response: Thank you. We considered and critically reviewed recommended citations before deciding whether to cite them.

Author-initiated correction:

During our review process, we identified an oversight regarding one cross-registered trial (NCT01638585; 2010-023426-20) that was not reported in the PRISMA flow diagram. This trial had tabular summary results available in the EU Clinical Trials Register (EUCTR); however, the results were provided exclusively as a PDF rather than in a structured tabular format.

The trial was initially carried forward for analysis, as the SAE risk methodology was in an iterative and novel development process. However, we later finalized the decision to exclude it because the comparability of PDF-based tabular results with structured tabular registry data in the EUCTR registry could not be ensured. Consequently, this trial was excluded from the final SAE analysis methodology. However, this exclusion was not accounted for in Step 1 (ideally would have been excluded here) or Step 2 (now excluded at this step) of the analysis according to the PRISMA flow diagram.

We reflect this change in the manuscript as follows:

• The following sentence has been added to the main manuscript: “During iterative development of the SAE analysis methodology, 1 cross-registered trial (NCT01638585, 2010-023426-20) was identified with summary results reported in tabular format in the EUCTR registry; however, these results were provided exclusively as a PDF document rather than in a structured tabular format. Because the comparability of PDF-based results with structured tabular results reported in the EUCTR registry could not be ensured, this trial was excluded in the final step.”

• The PRISMA flow diagram reflecting this change has also been updated. Additionally, a note has been attached to the diagram.

• The preprint has also been updated to reflect the changes made in the PRISMA flow diagram, with the note attached.

The final number of trials included (n = 14) in the SAE risk sub-analysis remains unchanged, and all analyses and conclusions are unaffected. The SAE risk methodology was developed iteratively, and trials were excluded at different stages during manual verification; this process is now explicitly described in the manuscript for transparency.

Responses to the comments made by Reviewer 1:

Comment: Please define cleary the eligibility criteria used in CT.gov registry. It is not defined did you retrieved trials with or without results, and which phases are included. Please clearly define that in “Eligibilty criteria” section” and in the data in OSF platform. The inclusion criteria should be defined following the filters in the ClinicalTrials.gov registry. Exclusion criteria is not defined also. This should be defined in the part “Eligibility criteria” section of manuscript.

Response: Thank you for this comment. We have revised the “Eligibility criteria” section to clearly define the eligibility criteria using filters from the ClinicalTrials.gov registry. This is also reflected on the OSF platform.

The following changes have been made:

“For both cohorts, inclusion criteria for this study were defined as follows:

Registry: Trials registered on ClinicalTrials.gov.

Recruitment status: Trials with recruitment status of ‘terminated’ in the ClinicalTrials.gov historical version dated December 1, 2023 were eligible. This version was downloaded using the cthist R package (17). Terminated trials are defined by ClinicalTrials.gov as studies in which recruitment or enrollment of participants was halted prematurely, participants were no longer being examined or receiving intervention, and the trial would not resume. Trials with a status of completed, defined as studies that concluded as planned with all participants having completed their final visits, were included for comparison (18).

Participants: At least one participant enrolled.

Intervention: All intervention types were included.

Trial study phase: All phases were included.”

In addition to rewriting this section, we also updated the “Data Sources” section to clarify how the cohorts were generated in the original studies, with their reference.

Comment: Differences in the ClinicalTrials.gov and other registries in the variety of trials should be presented as a limitation and explaines in that part of manuscript.

Response: Thank you for this very relevant comment. Trials conducted in Germany are often registered in multiple registries, including the EU Clinical Trials Register (EUCTR), ClinicalTrials.gov or the German Clinical Trials Register (DRKS). Medicinal product trials in the EU are legally required to be registered in the EUCTR (now transitioning to Clinical Trials Information System, CTIS). Many German trials registered in ClinicalTrials.gov are internationally conducted studies in which Germany is only one of multiple study sites. Registration in DRKS is non-mandatory, and it was also not the focus of our study. We used ClinicalTrials.gov and EUCTR registries to capture information on summary result reporting that might be missed if only a single registry had been considered (we viewed this as a strength). However, differences in reporting practices and in how data are collected and presented across registries limited the scalability of our methods. Nevertheless, we appreciate the reviewer’s suggestion and have added the following statement to the Limitations section:

“Additionally, differences between ClinicalTrials.gov and other trial registries in terms of the types of trials included, the structure of recorded information, and reporting formats may limit the generalizability and scalability of our methods.”

Responses to the comments made by Reviewer 2:

Comment: Thank you for the opportunity to review this interesting article about terminated trials from German and Californian university medical centers identified via ClinicalTrials.gov and the German study registry.

The data analysis consists of two previously assembled cohorts of clinical trials registered in California and Germany. The study explores the characteristics of prematurely terminated clinical trials affiliated with university medical centers in aforementioned locations, focusing on developing scalable semi-automated methods for their assessment. The authors compare terminated trials to completed ones regarding characteristics like result reporting and therapeutic focus. A secondary, exploratory aim was to estimate the risk of experiencing serious adverse events (SAEs) in trials terminated for non-scientific reasons.

Overall, the manuscript is well written, minor issues that could be addressed are as follows:

- Centres and centers could be written consistently in one form or the other.

- Non-scientific and nonscientific should be written consistently

Response: Thank you for the kind words. We have rechecked the manuscript for consistency in terminology and standardized the use of terms, including references to centers and non-scientific reasons for trial termination.

Minor issues:

Comment: Abstract: In the abstract, the authors describe the usage of the EUCTR as a data source in case of missing data in the ClinicalTrials register for the German studies; however, the authors do not mention that one primary data source for the German studies seems to be the DRKS database. If this is correct, please mention the database in the Abstract section; if it is not correct, please correct the methods so abstract and method sections are consistent.

Response: Thank you for this comment. The primary registry used for the German cohort in our analysis was ClinicalTrials.gov, not DRKS. Although DRKS has become increasingly important and is now the primary registry of choice for trials conducted in Germany, many German principal investigators historically registered their trials on ClinicalTrials.gov, particularly for internationally conducted studies. In addition, trials conducted in Germany are often registered in both ClinicalTrials.gov and EUCTR: Medicinal product trials in the EU are legally required to be registered in the EUCTR (now transitioning to Clinical Trials Information System, CTIS), while ClinicalTrials.gov includes many multinational studies in which Germany is only one of multiple study sites. Moreover, the tool used in our study was primarily designed for ClinicalTrials.gov data, which influenced our choice of primary data source.

To avoid confusion between the Abstract and the Methods sections, the manuscript refers only to ClinicalTrials.gov and EUCTR as data sources in the Abstract. DRKS is now mentioned only in the Methods section for the purpose of defining the original German cohort, but it was not used as a primary data source for the analyses reported in this study.

Major issues:

Comment: The manuscript states that publications for both cohorts were ‘searched and validated manually’, but no information is provided on how this search was conducted (databases used, search strings, matching criteria, number of reviewers, or cutoff date). As the analysis relies on publication detection originating from earlier cohort studies, the manuscript should summarise the original search methodology to ensure reproducibility and assess potential differences in search sensitivity between the German and Californian datasets.

Response: Thank you for this comment. Along with referencing the studies which describe the publication search methodology in detail, we have added the following statement to the main text:

“For both cohorts, publications reporting trial results were identified manually, either through links in the registry or, if no links were available, via Google searches using the trial ID or other matching parameters. A detailed overview of the search methodology can be found in the original studies. These datasets were selected due to their public availability and prior manual identification of results publication.”

Comment: What was the reason the authors used a different time interval as a comparison between the German cohort (2009-2017) and the Californian cohort (2014-2017); this introduces a potential bias in the comparison and should be discussed.

Response: Thank you for this comment, which was also very intensely discussed among coauthors during the course of study. The two cohorts were not intended to be compared directly across time. The selected time intervals reflect the completion periods of the original cohorts as recorded in the registries. All primary analyses were conducted within cohorts, comparing terminated and completed trials from the same registry and time period, rather than between cohorts. We agree that the use of different time intervals could otherwise be misinterpreted as introducing bias in cross-cohort comparisons. To prevent this unintended interpretation, we carefully reviewed the manuscript to ensure that no direct or indirect comparisons between cohorts were implied and added a clarifying statement to the Limitations section of the Discussion.

“The two cohorts were used to test the methodology and were drawn from different calendar periods based on data availability. To avoid potential bias, we ensured that all primary analyses were conducted within cohorts only, comparing terminated and completed trials from the same cohort and time period, and no direct comparisons were made between cohorts. We caution readers against drawing direct differences between cohorts.

Comment: In general, potential biases and weaknesses of the study approach should be discussed in more detail: Slow enrollment leading to trial termination might both be due to (underlying) business reasons; e.g., insufficient financial incentives for the medical centers to recruit patients or due to low efficacy and/or toxicity. I.e., toxicity or low efficacy in the first few patients recruited by a center or physician could lead to reluctance by physicians to recruit further patients. Therefore business reasons and scientific reasons might overlap in many cases and it is not possible to systematically account for these issues.

Response: Thank you for this comment. We agree that the way information is entered into trial registries may introduce ambiguity. In response, we have added the following statement to the Limitations section:

“In addition, reasons for trial termination were annotated solely based on information provided in the registries. Misclassification may thus arise from incomplete or ambiguous entries or from how information is recorded due to word limits. For example, “slow accrual” may reflect both business-related factors (e.g., insufficient financial incentives) and scientific considerations (e.g., early signals of limited efficacy or unacceptable toxicity). As a result, business and scientific reasons for termination may overlap and cannot be systematically disentangled using registry data alone”.

Comment: Similarly, excluding journal articles as a source for SAE reporting introduces a systematic bias: Data on inconsistent reporting as cited by the authors are credible in cases in which there is both a ClinicalTrials information (on SAE) available and a publication. However, excluding trials from such an analysis in which the ClinicalTrials data were unavailable but a full Journal article introduces another bias as there might be a systematic difference between trial sponsors who report results on ClinicalTrials and sponsors who are more reluctant (but would publish results in journal articles). As an example: Industry sponsors reported on trials more promptly and adhered closer to legal obligations than academic trials (Anderson et al. NEJM 2015). An academic sponsor might be interested in a publication while an update on ClinicalTrials might be sufficient for an industry sponsor if there is no commercial viability of the tested approach.

Response: This is an important point that was discussed during the development of our SAE risk methodology. During pilot testing, we found that adverse event reporting varies considerably between publications. Examples include: reporting only adverse events (AEs), reporting only treatment-related AEs, or discrepancies in total numbers across sources.

For this, having a standardized reporting format and comparable data was crucial. In light of this heterogeneity, developing an automated approach to reliably extract SAE data from journal articles is currently

Attachment Submitted filename: Response to Reviewers.docx

# Peer review: Decision Letter 1

**DOI:** 10.1371/journal.pone.0346608.r003

**Authors:** Sascha Köpke

23 Mar 2026

Exploring scalable assessment methods for terminated trials in ClinicalTrials.gov: A cohort analysis of German and Californian trials

PONE-D-25-38266R1

Dear Dr. Yerunkar,

We’re pleased to inform you that your manuscript has been judged scientifically suitable for publication and will be formally accepted for publication once it meets all outstanding technical requirements.

Within one week, you’ll receive an e-mail detailing the required amendments. When these have been addressed, you’ll receive a formal acceptance letter and your manuscript will be scheduled for publication.

An invoice will be generated when your article is formally accepted. Please note, if your institution has a publishing partnership with PLOS and your article meets the relevant criteria, all or part of your publication costs will be covered. Please make sure your user information is up-to-date by logging into Editorial Manager at Editorial Manager® and clicking the ‘Update My Information’ link at the top of the page. For questions related to billing, please contact  and clicking the ‘Update My Information’ link at the top of the page. For questions related to billing, please contact  and clicking the ‘Update My Information’ link at the top of the page. For questions related to billing, please contact  and clicking the ‘Update My Information’ link at the top of the page. For questions related to billing, please contact billing support....

If your institution or institutions have a press office, please notify them about your upcoming paper to help maximize its impact. If they’ll be preparing press materials, please inform our press team as soon as possible -- no later than 48 hours after receiving the formal acceptance. Your manuscript will remain under strict press embargo until 2 pm Eastern Time on the date of publication. For more information, please contact onepress@plos.org.

Kind regards,

Sascha Köpke

Academic Editor

PLOS One

# Editor report: Acceptance letter

**DOI:** 10.1371/journal.pone.0346608.r004

**Authors:** Sascha Köpke

PONE-D-25-38266R1

PLOS One

Dear Dr. Yerunkar,

I’m pleased to inform you that your manuscript has been deemed suitable for publication in PLOS One. Congratulations! Your manuscript is now being handed over to our production team.

At this stage, our production department will prepare your paper for publication. This includes ensuring the following:

* All references, tables, and figures are properly cited

* All relevant supporting information is included in the manuscript submission,

* There are no issues that prevent the paper from being properly typeset

You will receive further instructions from the production team, including instructions on how to review your proof when it is ready. Please keep in mind that we are working through a large volume of accepted articles, so please give us a few days to review your paper and let you know the next and final steps.

Lastly, if your institution or institutions have a press office, please let them know about your upcoming paper now to help maximize its impact. If they’ll be preparing press materials, please inform our press team within the next 48 hours. Your manuscript will remain under strict press embargo until 2 pm Eastern Time on the date of publication. For more information, please contact onepress@plos.org.

You will receive an invoice from PLOS for your publication fee after your manuscript has reached the completed accept phase. If you receive an email requesting payment before acceptance or for any other service, this may be a phishing scheme. Learn how to identify phishing emails and protect your accounts at https://explore.plos.org/phishing.

If we can help with anything else, please email us at customercare@plos.org.

Thank you for submitting your work to PLOS ONE and supporting open access.

Kind regards,

PLOS ONE Editorial Office Staff

on behalf of

Professor Sascha Köpke

Academic Editor

PLOS One
